# Reevaluating cirrhotic cardiomyopathy diagnostics

**DOI:** 10.1016/j.jhepr.2024.101052

**Published:** 2024-02-28

**Authors:** Benjamin Cailes, Omar Farouque, Avik Majumdar, Anoop N. Koshy

**Affiliations:** 1 The University of Melbourne Clinical School, Austin Health, Department of Cardiology, Melbourne, Victoria, Australia; 2 The University of Melbourne Clinical School, Austin Health, Victorian Liver Transplant Unit, Melbourne, Victoria, Australia

To the Editor:

We read with interest the recent review article by Liu *et al.*^1^ in which they explored the relative effectiveness of the two published guidelines for diagnosis of cirrhotic cardiomyopathy (CCM) and proposed the addition of a prolonged QT interval to these diagnostic criteria. While they should be commended for their summary of the extant literature in the field; we believe that there is additional literature that merits attention and which could further highlight the complexities of the CCM diagnosis.

Firstly, the authors highlight that patients with cirrhosis have hypercontractile left ventricular function at rest leading to a reduced contractile reserve, and concluded that there was currently a lack of standardized criteria to define this phenomenon, leading to its omission from the 2019 CCM diagnostic criteria.^1^^,^^2^ The fact that an increased cardiac output could be a surrogate for CCM and in turn its adverse sequelae at first seems counter intuitive; however, work from our group published subsequent to these criteria has provided further support for this hypothesis and proposed a new diagnostic criteria to better define this concept.^3^

We propose that an impaired increase in cardiac output of less than 25% upon low-dose dobutamine challenge, which correlates with a four-fold higher risk of hepatorenal syndrome, should be integrated into the diagnostic criteria.^2^^,^^3^ These findings support the hypothesis that it is a lack of contractile reserve on stress testing, rather than a reduced baseline left ventricular ejection fraction, which is a feature of CCM and work is ongoing to further investigate the use of this new diagnostic criterion as a potential diagnostic tool for the diagnosis of CCM that could contribute to future guidelines. Furthermore, while the addition of global longitudinal strain has been proposed in the diagnosis of CCM, the accuracy of global longitudinal strain to ascertain subclinical changes in cardiac function in patients with a hyperdynamic resting function is unclear.

Regarding the addition of a prolonged QT interval as a criterion for CCM, our findings suggest that, while it correlates with ventricular arrhythmia risk, it does not necessarily predict post-transplant complications or mortality.[4], [5], [6] There is also a lack of correlation between electrocardiographic and structural cardiac changes in cirrhosis, indicating distinct pathophysiological mechanisms for CCM and QT interval prolongation.^7^ This necessitates further exploration before its inclusion in diagnostic criteria.

Whilst we agree with the authors contention that the 2019 Cirrhotic Cardiomyopathy Consortium (CCC)^2^ guidelines appear to have better predictive and discriminatory ability than the previously proposed 2005 World Congress of Gastroenterology (WCG) guidelines, we put forward that there is certainly still scope to improve these criteria further. These criteria focus entirely on resting echocardiographic indices and the decision to omit any reference to an impaired contractile reserve appears to potentially be a significant one, as the hyperdynamic resting left ventricular function, which is often associated with more severe liver disease, likely represent patients encroaching on their cardiac reserve at rest. Further studies in this area are required to refine the diagnostic criteria for CCM and establish what cardiac testing is appropriate for establishing this diagnosis in the future.

## Financial support

The authors have not received any financial support for the production of this manuscript.

## Conflicts of interest

The authors have no conflicts of interest to report, excluding the fact that they have previously published in the area as referenced in the text.

Please refer to the accompanying ICMJE disclosure forms for further details.

## Authors’ contributions

BC, OF, AM and ANK all contributed to writing of the article.
